# Gα_o_ and Gα_q_ Regulate the Expression of *daf-7*, a TGFβ-like Gene, in *Caenorhabditis elegans*

**DOI:** 10.1371/journal.pone.0040368

**Published:** 2012-07-11

**Authors:** Edith M. Myers

**Affiliations:** Department of Biological and Allied Health Sciences, Fairleigh Dickinson University, College at Florham, Madison, New Jersey, United States of America; Brown University, United States of America

## Abstract

*Caenorhabditis elegans* enter an alternate developmental stage called dauer in unfavorable conditions such as starvation, overcrowding, or high temperature. Several evolutionarily conserved signaling pathways control dauer formation. DAF-7/TGFβ and serotonin, important ligands in these signaling pathways, affect not only dauer formation, but also the expression of one another. The heterotrimeric G proteins GOA-1 (Gα_o_) and EGL-30 (Gα_q_) mediate serotonin signaling as well as serotonin biosynthesis in *C. elegans*. It is not known whether GOA-1 or EGL-30 also affect dauer formation and/or *daf-7* expression, which are both modulated in part by serotonin. The purpose of this study is to better understand the relationship between proteins important for neuronal signaling and developmental plasticity in both *C. elegans* and humans. Using promoter-GFP transgenic worms, it was determined that both *goa-1* and *egl-30* regulate *daf-7* expression during larval development. In addition, the normal *daf-7* response to high temperature or starvation was altered in *goa-1* and *egl-30* mutants. Despite the effect of *goa-1* and *egl-30* mutations on *daf-7* expression in various environmental conditions, there was no effect of the mutations on dauer formation. This paper provides evidence that while *goa-1* and *egl-30* are important for normal *daf-7* expression, mutations in these genes are not sufficient to disrupt dauer formation.

## Introduction

Under unfavorable environmental conditions, developing *Caenorhabditis elegans* enter an alternative stage called dauer. In dauer, growth and feeding arrest. Dauer worms also have sealed orifices and form thickened cuticles. The metabolic and morphological changes that accompany dauer increase the likelihood of the animals’ survival under harsh conditions. The dauer stage is reversible, and larvae resume development when environmental conditions improve (reviewed in [1]).

Dauer formation is controlled in part by the DAF-7/TGFβ-like signaling pathway ([2] and reviewed in [1]). DAF-7 is expressed in the ASI sensory neurons and is required during larval development to inhibit dauer formation [3], [4]. Environmental cues such as starvation and high temperature that trigger dauer formation also downregulate *daf-7* expression [3]. While several genes are required for normal *daf-7* expression [5]–[7], the signaling pathways that control *daf-7* expression and its sensitivity to environmental signals are still not well understood.

One of the genes required for both *daf-7* expression and dauer formation encodes tryptophan hydroxylase, TPH-1 [6]. TPH-1 is the rate-limiting enzyme required for serotonin biosynthesis. Serotonin signals through the heterotrimeric G proteins GOA-1 and EGL-30 to control several *C. elegans* behaviors [8]–[11]. GOA-1 and EGL-30 share a high degree of homology with human Gα_o_ and Gα_q_ [12]. In the human nervous system, Gα_o_ and Gα_q_ act downstream of many neurotransmitters, including serotonin. In *C. elegans*, GOA-1 and EGL-30 also act upstream of *tph-1* to regulate its expression [13]. It is possible then, that *goa-1* and *egl-30* are important for regulating *daf-7* expression and dauer formation, and may do so by regulating either serotonin signaling or biosynthesis. These experiments explore, in a tractable model organism, a new relationship between evolutionarily conserved pathways and proteins important for neuronal signaling and developmental plasticity.

## Results and Discussion

### Gα_o_ and Gα_q_ do not Affect Morphology of *daf-7*-expressing Cells

**Figure 1:**
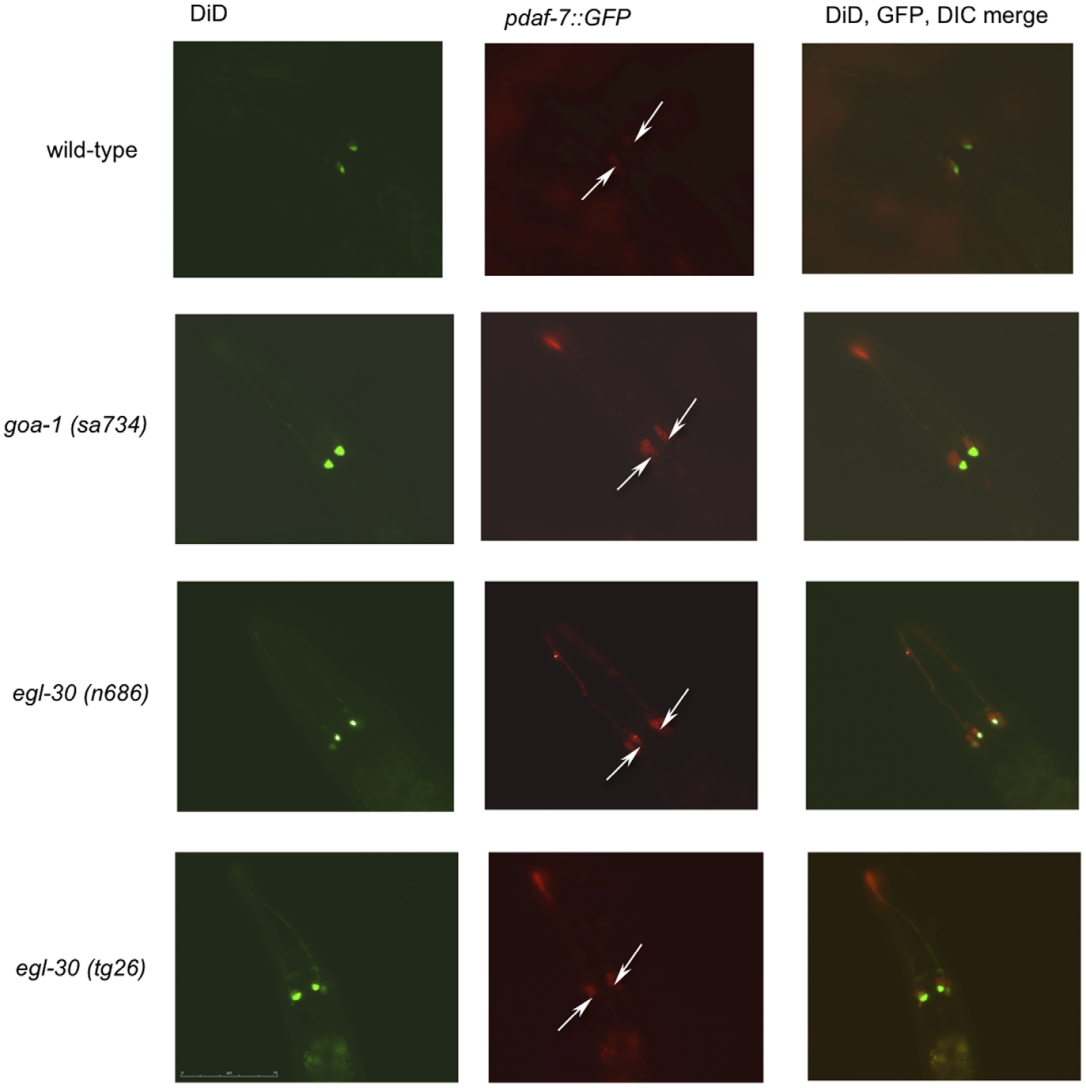
Mutations in *goa-1* and *egl-30* do not affect ASI development. Representative photomicrographs showing DiD filling (first column) and *pdaf-7*::GFP (second column) in wild type, *egl-30,* and *goa-1* mutant backgrounds. DiD filling was not significantly different between ASI neurons (indicated by white arrows) in wild type and mutant worms. ASI projections, visualized with *pdaf-7*::GFP, do not appear significantly altered in mutant worms. The last column shows a merge between DiD, GFP, and DIC images. *pdaf-7*::GFP strains used express GFP under the putative *daf-7* promoter, and only express GFP in the ASI neurons [5].

*daf-7* is expressed in two head sensory neurons called the ASIs [3], [4]. Structural changes in ASI cilia accompany dauer formation [14]. Two neuronal heterotrimeric G proteins, GPA-2 and GPA-3, affect dauer formation by affecting the sensory cilia of ASIs [15]–[17]. By altering the sensory cilia, GPA-2 and GPA-3 presumably affect the way *C. elegans* sense the environmental signals that regulate the dauer developmental switch. It is possible that additional G proteins such as GOA-1 and EGL-30 (which, unlike GPA-2 and GPA-3, have homologues in humans) could also primarily affect dauer formation or *daf-7* expression through altering the morphology of ASI. To first determine whether ASI morphology was affected by mutations in either *goa-1* or *egl-30*, gross neuronal structure was visualized using DiD. Worms were incubated in DiD, a lipophilic dye, that is only taken up by those sensory neurons making direct contact with the environment. Therefore, if ASI morphology were altered in either *goa-1* or *egl-30* mutants, the neurons would not fill with dye. ASIs in wild type, *goa-1 (n1134)* partial loss-of-function, *goa-1(sa734)* null, *egl-30* (*n686* and *ad805*) partial loss-of-function (*lf*) mutants as well as *egl-30 (tg26* and *js126*) gain-of-function (*gf*) mutants filled with DiD (Figure 1 and data not shown). These worms also exhibited normal gross morphology of ASIs, suggesting that signaling through either *goa-1* or *egl-30* is not required for ASI development.

### Gα_o_ and Gα_q_ are Required for *daf-7* expression but not Dauer Formation

**Figure 2:**
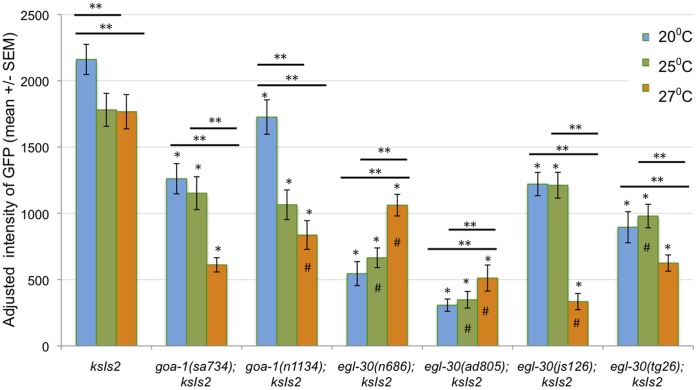
*goa-1* and *egl-30* regulate *daf-7* expression at multiple temperatures. *pdaf-7::*GFP levels were measured in late L1 larvae raised at 20°C, 25°C or 27°C. *goa-1* (*sa734 null* and *n1134lf*), *egl-30* (*n686lf* and *ad805lf*), and *egl-30* (*js126gf* and *tg26gf*) mutant larvae exhibited significantly less *daf-7* expression when compared to wild type larvae (*ksIs2*) raised at 20°C. *daf-7* expression was also significantly lower in all mutant larvae when compared to wild type larvae at both 25°C and 27°C. The decrease in *daf-7* in *goa-1(n1134lf*) and *egl-30*(*js126gf*) mutants was significantly greater than in wild type larvae raised at 27°C. *pdaf-7::*GFP expression actually increased as temperature increased to 25°C in *egl-30(n686lf*), *egl-30*(*ad805lf*) and *egl-30(tg26gf*) mutants, and increased again at 27°C in *egl-30(n686lf*) and *egl-30*(*ad805lf*) mutants. * =  significant difference from wild type (*ksIs2*) worms at the same temperature (Student’s t-test, p<0.05). ** =  significant difference between treatments in the same genotype (Student’s t-test, p<0.05). #  =  significant interaction between genotype and change in temperature, as compared to wild type, *ksIs2* larvae (ANOVA, p<0.05).

*daf-7* expression peaks during the first and second stages of larval development (L1 and L2; [3]) just before the dauer decision is made. When animals were raised in a favorable environment (with food at 20°C), all wild-type L1 larvae exhibited strong *daf-7* expression. *daf-7* expression was markedly reduced in larvae with (*lf)* alleles of *goa-1* or *egl-30* (Figure 2). In addition, *daf-7* expression was reduced in larvae with *egl-30 gf* alleles.

These data were unexpected for several reasons. First, one would expect that loss-of-function mutations and gain-of-function mutations in the same gene might have opposite effects on their target, in this case *daf-7*. However, both the (*lf)* and (*gf)* mutations in *egl-30* decreased *daf-7* expression.

**Figure 3:**
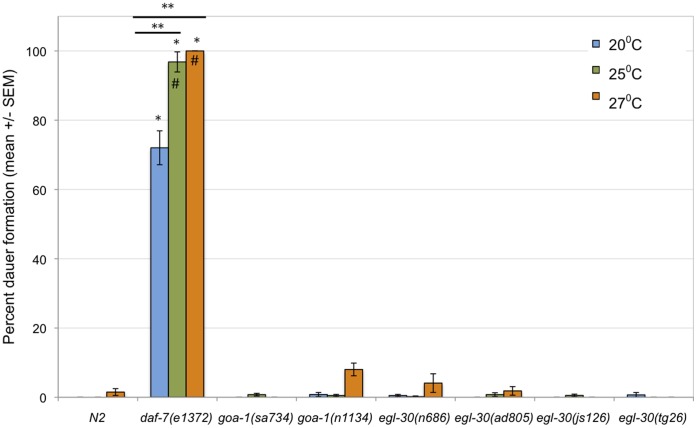
Neither *goa-1* nor *egl-30* inhibit dauer entry at high temperatures. Dauer formation was assayed in larvae raised at 25°C or 27°C. None of the mutant strains tested exhibited a significant increase in dauer formation at higher temperatures. It was not possible to assay dauer formation in *goa-1*(*sa734*) *null* or *egl-30 (tg26gf*) mutants at 27°C, because these mutants formed partial dauers or arrested at the L1 stage. Data for these strains at 27°C were therefore not included in the figure. *daf-7(e1372)* mutants are constitutive dauers at 25°C and 27°C, and served as a positive control. * =  significant difference from wild type (N2) worms at the same temperature (Student’s t-test, p<0.05). ** =  significant difference between treatments in the same genotype (Student’s t-test, p<0.05). #  =  significant interaction between genotype and change in temperature (from 20°C), as compared to wild type larvae (ANOVA, p<0.05).

The second unexpected result was that *daf-7* expression was reduced in both *goa-1* and *egl-30 (lf)* mutants. This was surprising because signaling through GOA-1 is thought to antagonize signaling through EGL-30. These two G proteins are thought to act antagonistically because they have opposite effects on many *C. elegans* behaviors [9], [18], [19]. In addition, *goa-1* and *egl-30* have opposite effects on *tph-1* expression; *goa-1* represses *tph-1* expression while *egl-30* promotes *tph-1* expression [13]. Since *tph-1* promotes *daf-7* expression [6], one would expect that *goa-1* (*lf)* mutations would cause an increase in *daf-7* expression while *egl-30 (lf)* mutations might case a decrease in *daf-7* expression. The data suggest that *goa-1* and *egl-30* are both required to maintain *daf-7* expression, and that any perturbation to either signaling pathway results in decreased *daf-7* expression. Because *goa-1* and *egl-30* are expressed in many cells throughout the worm, it is possible that *goa-1* and *egl-30* act in distinct subsets of cells that could have opposite effects on *daf-7* expression. For instance, *goa-1* could be required to activate a set of neurons that promotes *daf-7* expression, while *egl-30* could act to inhibit the activity of a set of neurons that inhibits *daf-7* expression. In a scenario such as this, (*lf*) mutations in both *goa-1* and *egl-30* would result in decreased *daf-7* expression. The decrease in *daf-7* expression seen in *goa-1* and *egl-30* mutants at 20°C was not sufficient, however, to elicit dauer formation (Figure 3).

How precisely EGL-30 and GOA-1 regulate *daf-7* expression may be difficult to elucidate because of a complex feedback loop that exists between *daf-7* and *tph-1*. While TPH-1 upregulates *daf-7* expression [6], DAF-7 downregulates *tph-1* expression [20]. *tph-1* expression is also elevated in dauer larvae [21] when *daf-7* expression is low. It is unlikely that GOA-1 or EGL-30 act downstream of *daf-7* to regulate *tph*-1 expression (and then *daf-7* expression) because *egl-30* and *goa-1* are not necessary for the increase in *tph-1* seen in dauer larvae [21]. GOA-1 and EGL-30 may instead be acting downstream or independently of *tph-1* to regulate *daf-7* expression.

### Gα_o_ and Gα_q_ Alter Temperature-induced Changes in *daf-7* Expression but not Dauer Formation

High temperatures can induce dauer formation in some mutants that do not readily form dauers at moderately high temperatures [22], [23]. It was possible that while *goa-1* and/or *egl-30* mutants did not form dauers at favorable temperatures (20°C, Figure 3), they would enter dauer at high temperatures. Moderately high (25°C) or high (27°C) temperatures were both insufficient to induce dauer formation in any of the G protein mutants tested (Figure 3). While most non-dauer worms developed into full adults, *goa-1(sa734) null* and *egl-30(tg26gf*) mutants did not. These non dauer worms appeared to arrest as larvae; either L1 or partial dauers (determined by SDS sensitivity). The larval arrest in these mutants occurred prior to the time in development when the dauer decision is made, and suggests that normal development at 27°C was disrupted by the *sa734* and *tg26* alleles.

Despite the absence of dauer formation seen at 25°C or 27°C, *daf-7* expression was significantly altered in all mutant strains tested (Figure 2). In almost all strains, there was a significant difference in the way temperature affected *daf-7* expression. These data suggest that GOA-1, and EGL-30 in particular, relay some sensory cues important for *daf-7* expression. EGL-30 appears to be important for downregulating *daf-7* in response to high temperatures. In fact, the effect of temperature on *daf-7* expression is reversed in the *egl-30(lf)* mutants and exaggerated in the *egl-30 (js126gf)* mutant. Other signaling pathways likely contribute to the behavioral/developmental response to high temperature since the decrease in *daf-7* expression in *egl-30* (*gf)* mutants was not sufficient to induce dauer formation at high temperatures.

### Gα_o_ and Gα_q_ Alter the Response to Limiting Amounts of Food

**Figure 4:**
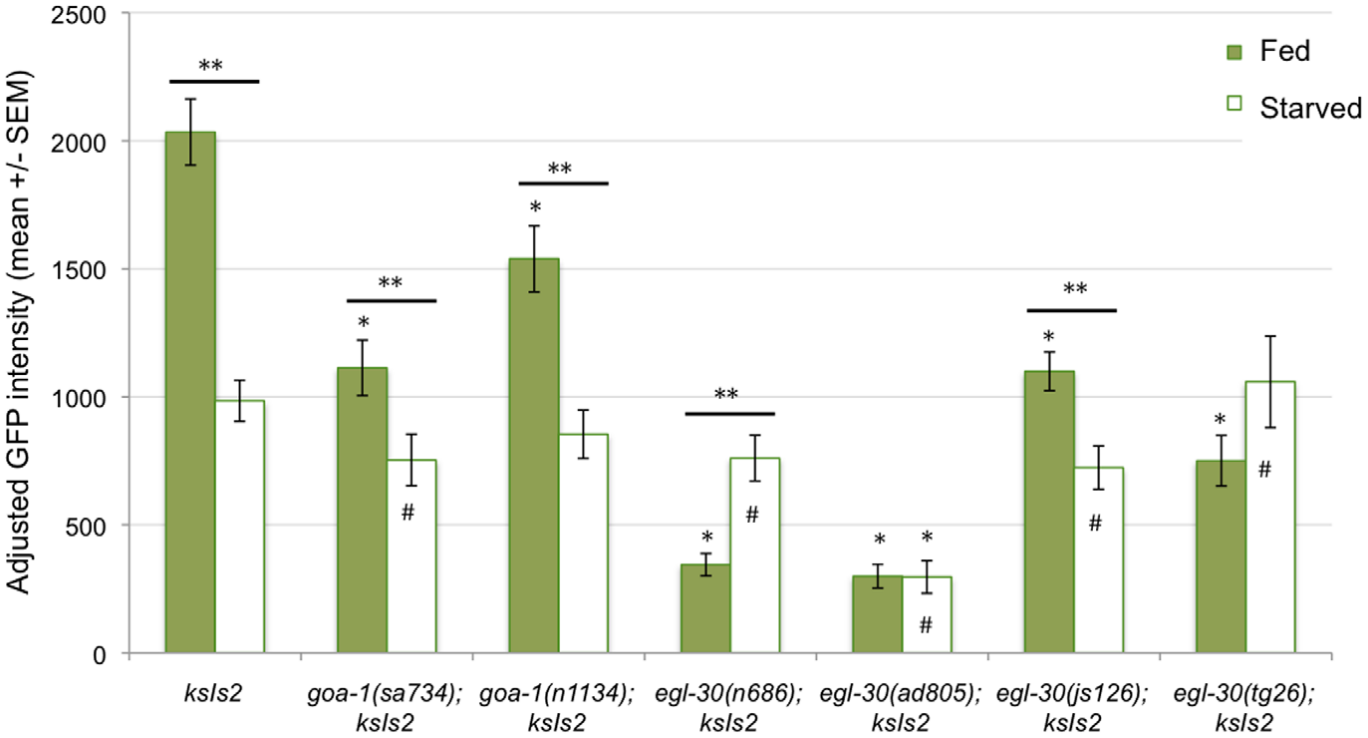
*goa-1* and *egl-30* regulate *daf-7* expression in response to starvation. *pdaf-7::*GFP levels were measured in well-fed late L1 larvae raised at 20°C or starved L1s. *daf-7* expression was lower with starvation in all genotypes except *egl-30(n686lf*), *egl-30* (*ad805lf*) and *egl-30(tg26gf*), in which *pdaf-7::*GFP expression was unchanged or increased with starvation. The decrease in *daf-7* in *goa-1(sa734*) *null*, and *egl-30 (js126gf*) mutants was significantly greater than in wild type larvae. * =  significant difference from wild type (*ksIs2*) worms at the same temperature (Student’s t-test, p<0.05). ** =  significant difference between treatments in the same genotype (Student’s t-test, p<0.05). #  =  significant interaction between genotype and change in temperature, as compared to wild type, *ksIs2* larvae (ANOVA, p<0.05).

In addition to temperature, food availability affects the course of *C. elegans* development. When larvae hatch from eggs in the absence of food, their development arrests at the L1 stage (prior to the time in development when the dauer decision is made) and *daf-7* expression is reduced in the arrested L1s [3]. Signaling through GOA-1 and EGL-30 modulates food sensitivity in adult *C. elegans* [24]–[26], so it is possible *goa-1* and *egl-30* are required for mediating the effect of food in larvae. As in wild-type L1s, starvation caused a significant reduction in *daf-7* in *goa-1* (*lf)* mutants (Figure 4). As expected, an equivalent reduction was seen in the *egl-30(tg26gf)* mutant and an increase was seen in the *egl-30(n686lf)* mutant. While these mutants still appeared to be in the arrested L1 stage, the data suggest that *egl-30* mediates the *daf-7* response to starvation. These results and those from studies in adult worms [25] suggest that EGL-30 plays the same role in the response to starvation throughout development.

Reduced *daf-7* expression was not seen in worms expressing the other *egl-30* (*gf)* mutant allele (*js126*), however. The difference between the *egl-30 (gf)* phenotypes may be caused by differences in the way each mutation affects the EGL-30 protein. The *tg26 (gf)* allele is thought to contain a mutation that alters guanine nucleotide binding [27]. The *js126* (*gf)* allele is thought to contain a mutation that alters GTPase activity [28]. Both *tg26* and *js126* mutants have (*gf)* phenotypes with respect to other EGL-30-dependent behaviors such as egg laying and movement [28], [29], however it is not clear whether it is reasonable to predict that both alleles would affect all ELG-30-dependent processes in the same way.

**Figure 5:**
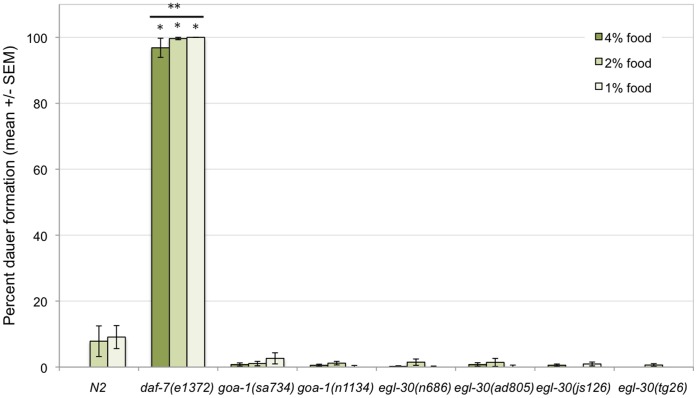
Neither *goa-1* nor *egl-30* are required for dauer formation in response to reduced food. Dauer formation was assayed in larvae grown at 25°C on several concentrations of *E. coli OP50*. Wild type larvae formed dauers as food concentration decreased. In at least one assay, non-dauer worms of *egl-30* (*tg26gf*), *egl-30* (*n686lf*), *egl-30* (*ad805lf*), or *goa-1* (*n1134lf*) genotype did not develop into adults when grown on 1% food. Instead, these larvae were arrested as L1s or partial dauers. Data for these strains at 1% food concentration were therefore not included in the figure. * =  significant difference from wild type (N2) worms at the same food concentration (Student’s t-test, p<0.05). ** =  significant difference between treatments in the same genotype (Student’s t-test, p<0.05).

When larvae hatch in the presence of limiting amounts of food, they progress through the L1 stage and then enter dauer [30]. If *egl-30* and *goa-1* are important for relaying the food cues important for normal development, one would expect that mutations in either *goa-1* or *egl-30* would disrupt dauer formation caused by low food levels. When *goa-1* and *egl-30* mutant larvae were exposed to limiting amounts of food, they did arrest at an early stage of development (Figure 5). However, based on size, most larvae appeared to be arrested at the L1 or partial dauer stages and not as full dauers. Most of the small larvae exhibited pharyngeal pumping and were sensitive to SDS, indicating that they did not arrest as full dauers [31]. These data suggest that *goa-1* and *egl-30* mutant worms are still sensitive to alterations in food availability, because they did not fail to arrest development in the presence of limiting food.

Overall, the experiments in this study showed that *goa-1* and *egl-30* regulate *daf-7* expression in early development. While *goa-1* and *egl-30* mutations significantly decreased *daf-7* expression, they did not affect dauer formation. These results suggest that other signaling pathways act in concert with GOA-1 and EGL-30 to decrease *daf-7* expression to levels sufficient to induce dauer formation.

## Materials and Methods

### Worm Strains

*C. elegans* worm strains were maintained on NGM plates with *Escherichia coli* OP50 as the food source [32]. Strains were provided by the Caenorhabditis Genetic Center (CGC) and were derived from the wild-type N2 Bristol strain. Strains used were as follows: N2, JT734 *goa-1(sa734),* KO96 *goa-1(n1134lf)*, MT1434 *egl-30(n686sd),* NM1380 *egl-30(js126gf),* KY26 *egl-30(tg26gf)*, DA823 *egl-30* (*ad805*), and CB1372 *daf-7(e1372ts).* Strains containing G protein mutations were crossed into the FK181 *ksIs2 [pdaf-7::GFP, rol-6(su1006)]* strain for *pdaf-7::GFP* analysis.

### Microscopy

For all assays, the developmental stages of larvae were carefully synchronized. For temperature assays, gravid adults laid eggs on NGM plates for 4 hours. Adults were removed and eggs were grown on NGM plates for 18–24 hours. For starvation assays, gravid adults were bleached to isolate eggs. Eggs were grown on NGM plates for 18–24 hours (fed) or in M9 medium for 48 hours (starved). Larvae were transferred to a 4% agarose pad on a microscope slide, immobilized with 10 mM levamisole, and viewed using a Leica DM5500 microscope. ASI images were captured with a fixed exposure time using a Hammamatsu Orca ER camera and Leica Microsystems Image capture software. GFP intensity was quantified using NIH Image J software version 1.44o. The intensity of GFP in each ASI cell body was quantified. The intensity of a similarly sized background selection was subtracted from the ASI GFP intensity to get the adjusted GFP intensity. Approximately ten larvae of each genotype were imaged in each experiment. Experiments were performed in triplicate, on three separate days. Dye filling was performed using 0.1 mg/ml DiD (Molecular Probes) as described [33].

### Dauer Assays

Dauer assays were done similarly to those previously described [30]. Modified NGM plates were prepared without peptone, and Noble agar (Difco) was used. 3 ml of modified NGM was used in each dauer assay plate. Plates were seeded with 20 µl of 4% (w/v) OP50, unless otherwise noted. *E. coli OP50* was resuspended in S Medium with 50 µg/ml streptomycin. Larvae were synchronized by first isolating eggs from bleached, gravid adults. Eggs were resuspended in S Medium. Approximately 100 eggs were pipetted onto seeded dauer plates that were incubated for 60–72 hours. Dauer worms were scored visually, and scoring was confirmed using SDS. Worms were considered dauers if they survived a several minute incubation in 1%SDS. Larvae were considered partial dauers if they were the same size and shape as dauer larvae, but exhibited pharyngeal pumping and did not survive SDS treatment.

Assays were performed with 100–200 worms for each genotype. All genotypes were tested in an assay. Assays were performed in triplicate, on three separate days.
